# Disrupting BMP/TGF‐β Signaling: Modulation of *AQP1* and *TGFB1* in Human Pulmonary Microvascular Endothelial Cells

**DOI:** 10.1002/cph4.70066

**Published:** 2025-10-29

**Authors:** Chrysi Keskinidou, Nikolaos S. Lotsios, Kostas A. Papavassiliou, Athanasios G. Papavassiliou, Ioanna Dimopoulou, Anastasia Kotanidou, David Langleben, Stylianos E. Orfanos, Alice G. Vassiliou

**Affiliations:** ^1^ First Department of Critical Care Medicine, School of Medicine National and Kapodistrian University of Athens, Evangelismos Hospital Athens Greece; ^2^ First University Department of Respiratory Medicine, ‘Sotiria’ Chest Hospital, School of Medicine National and Kapodistrian University of Athens Athens Greece; ^3^ Department of Biological Chemistry, School of Medicine National and Kapodistrian University of Athens Athens Greece; ^4^ Center for Pulmonary Vascular Disease, Azrieli Heart Center and Lady Davis Institute, Jewish General Hospital McGill University Montreal Quebec Canada; ^5^ School of Medicine National and Kapodistrian University of Athens Athens Greece

**Keywords:** AQP1, BMP9, BMPR2, PAH, TGF‐β

## Abstract

Pulmonary arterial hypertension (PAH) is a chronic disorder with high fatality rates, and its progression is highly associated with the genetic background. Alongside pathogenic variants in genes central to the BMP/TGF‐β signaling pathway, recent evidence has linked aquaporin 1 (*AQP1*) gene variants to PAH. While BMP9 shows promise as a PAH therapy, emerging conflicting evidence challenges this prospect. Herein, we modulated the gene expression of *AQP1* and *TGFB1* and examined their effect, before and after BMP9 administration, on BMP9, BMP10, BMPR2, AQP1, TGFBR1, and TGFB1 in human pulmonary microvascular endothelial cells (HPMECs) in vitro. Our results demonstrated that silencing of the *AQP1* gene resulted in decreased BMPR2 mRNA and protein, downregulated *TGFB1* and *TGFBR1* mRNA, while tending to reduce TGFBR1 protein levels. BMP9 exogenous administration affected only *TGFB1* mRNA, restoring control levels. Silencing of the *TGFB1* gene downregulated BMPR2 mRNA and protein levels and affected the expression of its ligands; BMP9 mRNA and protein increased, while *BMP10* mRNA levels decreased. Exogenous BMP9 treatment of *TGFB1*‐silenced cells decreased AQP1 mRNA and protein levels. Our results indicate that modulation of *AQP1* and *TGFB1* genes could possibly disrupt the complex signaling pathway, and that the effects of BMP9 may be cell‐ and context‐dependent. Together, these findings could provide a novel perspective on the interactions of the BMP/TGF‐β signaling pathway.

## Graphical abstract

Evaluation of targeted gene silencing and exogenous BMP9 administration in HPMECs. *AQP1* silencing decreased TGFB1, TGFBR1, and BMPR2 expression, while BMP9 administration returned TGFB1 to basal levels. *TGFB1* silencing upregulated BMP9, while downregulating BMP10 and BMPR2 expression. BMP9 administration downregulated AQP1 expression. These results support the context‐dependent effects of BMP9.

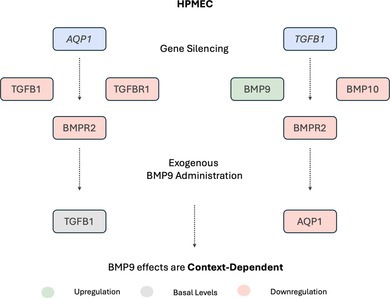

## 1 Introduction

Pulmonary arterial hypertension (PAH) is a progressive and often fatal disorder characterized by structural changes in the pulmonary vascular bed, including muscularization of pre‐capillary pulmonary arteries, formation of plexiform lesions, and increased pulmonary arterial pressure (Li and Quigley 2024). The pulmonary endothelium plays a central role in PAH pathogenesis (Huertas et al. 2018). Disease initiation likely stems from endothelial cell (EC) apoptosis, triggered by genetic susceptibility or injury (e.g., shear stress, chronic hypoxia, or inflammation) (Evans et al. 2021). Bone morphogenetic protein receptor type II (BMPR2) loss‐of‐function variants or deficient BMP9 signaling disrupt the BMP/transforming growth factor β (TGF‐β) balance, and the EC survival pathways (Long et al. 2015; Morrell et al. 2019). This selects for ECs with hyperproliferative, apoptosis‐resistant phenotypes, driving obstructive vascular remodeling (Marinho et al. 2024). Endothelial‐to‐mesenchymal transition (EndMT) may further amplify remodeling, though its clinical significance remains under investigation (Gorelova et al. 2021). Concurrent endothelial dysfunction, manifested by vasoconstriction, prothrombotic states, and impaired angiogenesis, propagates disease progression (Humbert et al. 2019; Cober et al. 2022).

Following oligomerization of the receptors upon ligand binding, signaling continues with phosphorylation of the cytoplasmic molecules, suppressors of mothers against decapentaplegic (SMAD)2/3 for the TGF‐β pathway, or SMAD1/5/9 for the BMP pathway. These then form a stable complex with the co‐mediator SMAD4. Finally, this complex translocates to the nucleus and regulates gene expression (Heldin and Moustakas 2012). Inhibitor of DNA binding proteins (ID1–4) are major downstream transcriptional targets of BMP signaling, while activation of SMAD2/3 leads to upregulated plasminogen activator inhibitor 1 (PAI‐1) transcription (Katta et al. 2008; Yang et al. 2010, 2013). Dysfunctional BMPR2‐dependent signaling pathways (Evans et al. 2016; Gräf et al. 2018) and overactive TGF‐β pathways are considered hallmarks of PAH (Gräf et al. 2018; Bousseau et al. 2023).

However, while pathogenic variants of genes involved in these signaling pathways represent a major causative factor in PAH, other genomic studies have identified novel genes potentially contributing to the disease. Recently, the task force of the 7th “World Symposium on Pulmonary Hypertension genetics and genomics” and the “Pulmonary Hypertension Gene Curation Expert Panel” published their conclusions and recommendations on the level of evidence supporting PAH gene‐disease associations. Among the genes associated with PAH, aquaporin 1 (*AQP1*) was classified as having limited evidence, which is consistent with the fact that only within the past 5 years has the *AQP1* gene been linked to PAH (Welch et al. 2023; Austin et al. 2024). AQP1 is a membrane protein within the aquaporin family, with a significant role in water transport regulation across the cell membrane (Lotsios et al. 2023). It has gained attention in the setting of PAH for its role in regulating endothelial cell permeability, migration, and proliferation (Meli et al. 2018). Notably, our group recently described a link between AQP1 and components of the BMP signaling pathway, namely BMPR2 and TGFΒ1 and demonstrated that in human pulmonary microvascular endothelial cells (HPMECs), silencing of either the *BMPR2* or the *AQP1* gene results in decreased expression of *TGF* *Β* *1* (Vassiliou et al. 2020, 2021).

Elucidating the genetic landscape of PAH could represent potential targets for the development of effective therapeutic strategies (Adu‐Amankwaah et al. 2025). The traditional PAH treatments primarily target the endothelin 1, nitric oxide, and prostacyclin pathways to control pulmonary vascular tone and proliferation. Although these therapies improved the patients' symptoms and reduced the risk of deterioration, unfortunately, they have not yet provided a cure (Li and Quigley 2024). Among the novel therapeutic strategies explored, the therapeutic administration of recombinant BMP9, the main ligand of BMPR2, has been presented as a potential strategy for enhancing endothelial BMP signaling in PAH (Long et al. 2015). However, the downstream effects of exogenous BMP9 administration on BMP signaling molecules could depend on the expression levels of BMPR2 (Lotsios et al. 2024). Recently, sotatercept, an activin‐binding molecule, has demonstrated significant effectiveness and has received approval for clinical use. Unlike previous therapies, sotatercept does not target the vascular tone, but partially restores the BMPR2/TGF‐β signaling imbalance and appears to be involved in reversing the remodeling seen in PAH (Preston et al. 2024). It becomes evident that fully elucidating the underlying molecular interactions within these pathways is crucial.

It remains unclear whether the presence of co‐occurring genetic variants or the interaction between proteins involved in the BMP signaling pathway is responsible for the establishment of PAH. To date, genetic studies have identified several pathogenic variants as causal factors for the development of PAH; however, there are no studies examining the interaction of the proteins involved. In this study, we examined the effect of exogenous administration of BMP9 in regulating the BMP/TGF‐β pathways in *AQP1*‐ and *TGFB1*‐silenced HPMECs, aiming to understand their involvement in the pathological mechanisms in PAH.

## 2 Materials and Methods

### 2.1 Cell Culture, Transfection, and Treatment With BMP9

All experiments were performed using the HPMEC‐ST1.6R cell line, originated from normal lung tissue distant from the tumor site of a 63‐year‐old male donor (Krump‐Konvalinkova et al. 2001). Cell transfection for the two siRNAs was carried out as previously described in detail (Lotsios et al. 2024). HPMECs were treated with BMP9 (5 ng/mL) (OriGene, Rockville, MD, USA) 24 h post‐transfection. Following a further 24 h incubation, cells were harvested for total RNA and total protein extraction.

### 2.2 RNA and Protein Extraction

Total RNA and total protein were extracted 48 h post‐transfection from both BMP9‐treated and untreated cells. Extraction was performed on cells cultured in the same plate to ensure transfection efficiency. Total RNA was extracted using the TRI reagent (Thermo Fisher Scientific, Waltham, MA, USA), according to the manufacturer's instructions. The concentration and quality of the total extracted RNA were determined at 260 and 280 nm, using the Nanodrop spectrophotometer (Thermo Fisher Scientific, Waltham, MA, USA). For protein extraction, samples were sonicated with an ultrasonic lab homogenizer (Thermo Fisher Scientific, Waltham, MA, USA), following pellet resuspension in lysis buffer. Total protein concentration was determined using the bicinchoninic acid (BCA) method (Lleu and Rebel 1991) (Thermo Fisher Scientific, Waltham, MA, USA).

### 2.3 Reverse Transcription and Quantitative Real‐Time PCR

From each sample, 100 ng of total RNA were reverse transcribed into single‐stranded cDNA (Nippon Genetics, Duren, Germany), following the manufacturer's instructions. Quantitative real‐time polymerase chain reaction (qPCR) method was employed, using Kapa SYBR Green PCR Master Mix (Sigma‐Aldrich, St Louis, MO, USA), to measure *AQP1*, *BMP9*, *BMP10*, *BMPR2*, *ID1*, *PAI1 (SERPINE1)*, *SMAD2*, *SMAD3*, *SMAD4*, *TGFB1*, *TGFBR1*, cyclophilin A (*CYPA*) and *GAPDH* mRNA expression levels. The analysis was carried out on a CFX Connect thermocycler (Bio‐Rad Laboratories). The specific primer sets designed for the targeted genes are listed in Table 1. Non‐transfected control cells were used as a calibrator, and the relative gene expression levels were determined through the comparative CT method 2^−ΔΔCT^ (Livak and Schmittgen 2001). Expression of the *CYPA* or *GAPDH* housekeeping genes was used for normalization purposes.

**TABLE 1:** Gene‐specific primer pairs used in quantitative real‐time PCR experiments.

Gene		Sequence (5′–3′)	nt
*AQP1*	F	5′‐TATGCGTGCTGGCTACTACCGA‐3′	22
	R	5′‐GGTTAATCCCACAGCCAGTGTAG‐3′	23
*BMP9*	F	5′‐CCTGCCCTTCTTTGTTGTCTTCTC‐3′	24
	R	5′‐TGACTGCTCTCACCTGCCTCTGTG‐3′	24
*BMP10*	F	5′‐AAGCCTATGAATGCCGTGGTG‐3′	21
	R	5′‐AGGCCTGGATAATTGCATGCTT‐3′	22
*BMPR2*	F	5′‐CCACCTCCTGACACAACACC‐3′	20
	R	5′‐TGTGAAGACCTTGTTTACGGT‐3′	21
*CYPA*	F	5′‐GCTGGACCCCTCAGGCATTT‐3′	20
	R	5′‐TTGCCAAACACCACATGCTT‐3′	20
*GAPDH*	F	5′‐ATGGGGAAGGTGAAGGTCG‐3′	19
	R	5′‐GGGGTCATTGATGGCAACAATA‐3′	22
*ID1*	F	5′‐CTGCTCTACGACATGAACGGC‐3′	21
	R	5′‐TGACGTGCTGGAGAATCTCCA‐3′	21
*PAI1 (SERPINE1)*	F	5′‐GGCTGACTTCACGAGTCTTTCA‐3′	22
	R	5′‐TTCACTTTCTGCAGCGCCTA‐3′	20
*SMAD2*	F	5′‐CGTCCATCTTGCCATTCACG‐3′	20
	R	5′‐CTCAAGCTCATCTAATCGTCCTG‐3′	23
*SMAD3*	F	5′‐GCGTGCGGCTCTACTACATC‐3′	20
	R	5′‐GCACATTCGGGTCAACTGGTA‐3′	21
*SMAD4*	F	5′‐GCTGCTGGAATTGGTGTTGATG‐3′	22
	R	5′‐AGGTGTTTCTTTGATGCTCTGTCT‐3′	24
*TGFB1*	F	5′‐GCGTGCTAATGGTGGAAAC‐3′	19
	R	5′‐CGGTGACATCAAAAGATAACCAC‐3′	23
*TGFBR1*	F	5′‐GACAACGTCAGGTTCTGGCTCA‐3′	22
	R	5′‐CCGCCACTTTCCTCTCCAAACT‐3′	22

Abbreviations: AQP1, aquaporin 1; BMP, bone morphogenetic protein; BMPR2, bone morphogenetic protein receptor type II; CYPA, cyclophilin A; GAPDH, glyceraldehyde‐3‐phosphate dehydrogenase; ID1, inhibitor of DNA binding 1; PAI1, plasminogen activator inhibitor 1; SMAD, suppressors of mothers against decapentaplegic; TGFB1, transforming growth factor beta 1; TGFBR1, transforming growth factor beta receptor I.

### 2.4 SDS‐Polyacrylamide Gel Electrophoresis (PAGE) and Immunoblotting

SDS–PAGE was performed using polyacrylamide slab gels on a “Biorad Mini Pro‐tean II” electrophoresis apparatus (Bio‐Rad Laboratories Inc., Hercules, CA, USA), as previously described (Laemmli 1970). Subsequently, samples were transferred onto a 0.45 μΜ pore size Immobilon‐P PVDF membrane (MilliporeSigma, Burlington, MA, USA), using a wet transfer apparatus (Bio‐Rad Laboratories Inc., Hercules, CA, USA). Immunological detection followed using specific antibodies for AQP1 and BMP9 (MilliporeSigma, Burlington, MA, USA), BMP10 and TGFB1 (Affinity Biosciences, Cincinnati, OH, USA), BMPR2, phosphorylated SMAD2 (p‐SMAD2), and TGFBR1 (Cell Signaling Technology, MA, USA). Actin (MilliporeSigma, Burlington, MA, USA) and β‐tubulin (Santa Cruz Biotechnology, TX, USA) were used as loading controls. Relative protein expression was estimated by densitometry with the use of the iBright Analysis Software (Thermo Fisher Scientific, Waltham, MA, USA).

### 2.5 Statistical Analysis

Data are presented as box plots, presenting median values with interquartile range (IQR), or as bar plots with mean ± SEM. The non‐parametric Mann‐Whitney test or the student's *t*‐test were employed for statistical analysis, as appropriate. All tests were carried out using the GraphPad Prism 9 software (GraphPad Software, San Diego, CA, USA). All *p*‐values are two‐sided; statistical significance was set at *p* < 0.05.

## 3 Results

### 3.1 Effects of *AQP1* ‐Silencing and Exogenous Administration of BMP9 on AQP1 and BMP/TGF‐β Signaling Molecules in Human Pulmonary Microvascular Endothelial Cells

**FIGURE 1:**
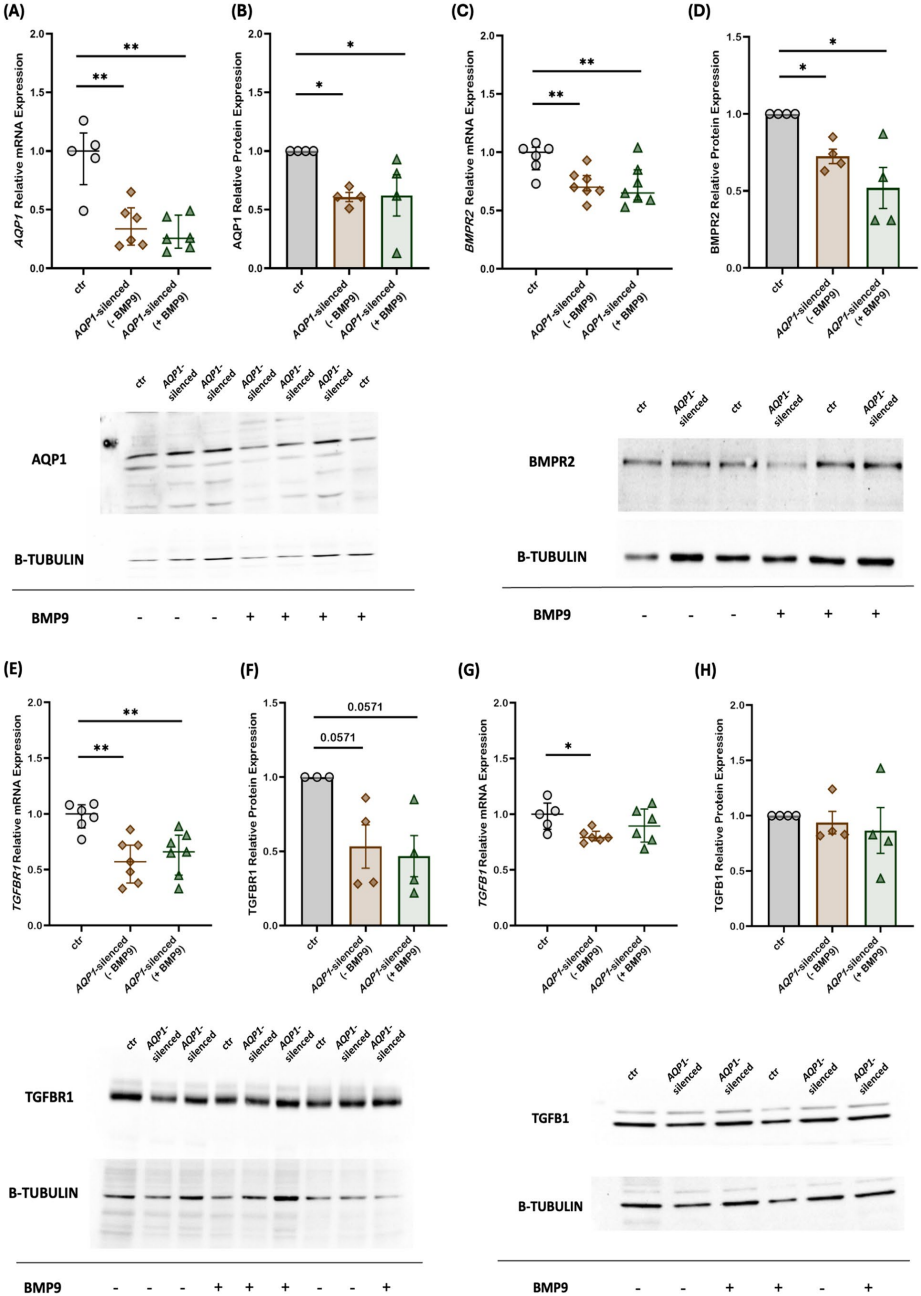
Effects of silencing the *AQP1* gene and exogenous administration of BMP9 on AQP1 and BMP/TGF‐β signaling molecules in human pulmonary microvascular endothelial cells. HPMECs were silenced for the *AQP1* gene and the relative mRNA and protein expression of AQP1 (A, B), BMPR2 (C, D), TGFBR1 (E, F), and TGFB1 (G, H) were estimated before and after the exogenous administration of BMP9. Relative mRNA expression is shown in dot plots (dots, individual values; line in the middle, median values; lower and upper lines, 25th and 75th percentiles) (A, *n* = 6; C, *n* = 7; E, *n* = 7; G, *n* = 6). Protein expression was analyzed by SDS‐PAGE and immunoblotting, and relative expression was estimated by densitometry using β‐tubulin as a loading control. Relative protein expression is shown with bar plots (mean ± SEM; dots, individual values) (B, *n* = 4; D, *n* = 4; F, *n* = 4; H, *n* = 4). Representative expression of AQP1 (B), BMPR2 (D), TGFBR1 (F), and TGFB1 (H) (upper panels) and β‐tubulin (lower panels) proteins in the *AQP1*‐silenced HPMEC homogenates. Specificity (siRNA negative control), efficiency (*AQP1* siRNA), and the effect of BMP9 exogenous administration on the non‐transfected controls were tested each time to ensure the consistency and reproducibility across the independent experiments. Statistical analysis was performed using the Mann–Whitney test. *, *p* < 0.05; **, *p* < 0.01 compared to the non‐transfected control HPMECs.

Initially, our experimental model was tested for transfection specificity and efficiency employing quantitative PCR. HPMECs were transfected using a universal scrambled negative control siRNA duplex (siRNA controls). Relative *AQP1* mRNA expression levels in the siRNA controls (0.64 [0.45–0.90]) did not differ from those of the non‐transfected control cells (1.00 [0.72–1.16]) (*p* > 0.05). Cell transfection with the *AQP1*‐specific siRNA resulted in a significant decrease in *AQP1* mRNA expression compared to the non‐transfected control cells (0.34 [0.20–0.52] vs. 1.00 [0.72–1.16], respectively, *p* < 0.01; Figure 1A). Exogenous administration of BMP9 failed to restore the reduced *AQP1* mRNA levels observed in the *AQP1*‐silenced cells (0.26 [0.17–0.45], *p* < 0.01, compared to the non‐transfected control cells; Figure 1A). Regarding AQP1 protein levels, silencing of the *AQP1* gene resulted in decreased protein levels compared to the non‐transfected control cells (0.61 ± 0.04, *p* < 0.05; Figure 1B), while BMP9 administration did not reverse this effect on AQP1 protein levels (0.62 ± 0.18, *p* < 0.05; Figure 1B compared to the non‐transfected control cells).

In previously published research, we have demonstrated the effect of *AQP1*‐silencing on *BMPR2* mRNA expression (Vassiliou et al. 2021). At first, we reconfirmed our findings demonstrating that silencing of the *AQP1* gene results in decreased *BMPR2* mRNA expression compared to non‐transfected control cells (0.70 [0.67–0.80] vs. 1.00 [0.85–1.04], respectively, *p* < 0.05; Figure 1C). *BMPR2* mRNA expression remained low even after the exogenous administration of BMP9 (0.65 [0.59–0.85], *p* < 0.05, compared to the non‐transfected control cells; Figure 1C). BMPR2 protein levels demonstrated a similar pattern to that of mRNA. *AQP1*‐silenced cells presented decreased BMPR2 protein expression compared to the non‐transfected control cells (0.73 ± 0.05, *p* < 0.05; Figure 1D). BMPR2 levels remained stable in the *AQP1*‐silenced cells following exogenous BMP9 administration (0.52 ± 0.13, *p* < 0.05, compared to the non‐transfected control cells; Figure 1D). Moreover, the mRNA expression levels of *ID1* in the *AQP1*‐silenced cells were decreased compared to the non‐transfected control cells (0.46 [0.35–0.83] vs. 1.01 [0.75–1.53], respectively, *p* < 0.05; Figure S1A).

Next, we proceeded to evaluate the effect of silencing the *AQP1* gene on both TGFBR1 and TGFB1 expression, a key receptor‐ligand duo in the BMP/TGF‐β signaling pathway. *TGFBR1* mRNA expression decreased significantly in comparison to the non‐transfected control cells (0.57 [0.38–0.72] vs. 1.00 [0.88–1.08], respectively, *p* < 0.01; Figure 1E). Exposure to exogenous BMP9 did not affect *TGFBR1* mRNA expression in the *AQP1*‐silenced cells compared to the non‐transfected control cells (0.66 [0.45–0.81], *p* < 0.01; Figure 1E). A similar trend was discovered for the TGFBR1 protein levels, although the statistical significance was marginal. More specifically, in *AQP1*‐silenced cells, TGFBR1 protein levels were decreased compared to the non‐transfected control cells both pre‐ and post‐exogenous BMP9 administration (0.53 ± 0.15, *p* = 0.057; Figure 1F and 0.47 ± 0.14, respectively, *p* = 0.057; Figure 1F). A significant decrease in *TGFB1* mRNA expression was seen following *AQP1*‐silencing compared to the non‐transfected control cells (0.79 [0.76–0.85] vs. 1.00 [0.87–1.10], respectively, *p* < 0.05; Figure 1G). Administration of exogenous BMP9 restored *TGFB1* mRNA expression to levels comparable with the non‐transfected control cells (0.90 [0.75–1.05], *p* > 0.05; Figure 1G). TGFB1 protein levels following *AQP1*‐silencing did not differ compared to the non‐transfected control cells (0.94 ± 0.10, *p* > 0.05; Figure 1H). Exogenous administration of BMP9 did not alter TGFB1 protein levels in the *AQP1*‐silenced cells (0.87 ± 0.21, *p* > 0.05; Figure 1H). Finally, the mRNA expression levels of *PAI1* in the *AQP1*‐silenced cells were decreased compared to the non‐transfected control cells (0.58 [0.29–0.71] vs. 1.00 [0.59–1.12], respectively, *p* < 0.05; Figure S1B).

### 3.2 Effects of *TGFB1* ‐Silencing and Exogenous Administration of BMP9 on BMP/TGF‐β Signaling Molecules and AQP1 in Human Pulmonary Microvascular Endothelial Cells

**FIGURE 2:**
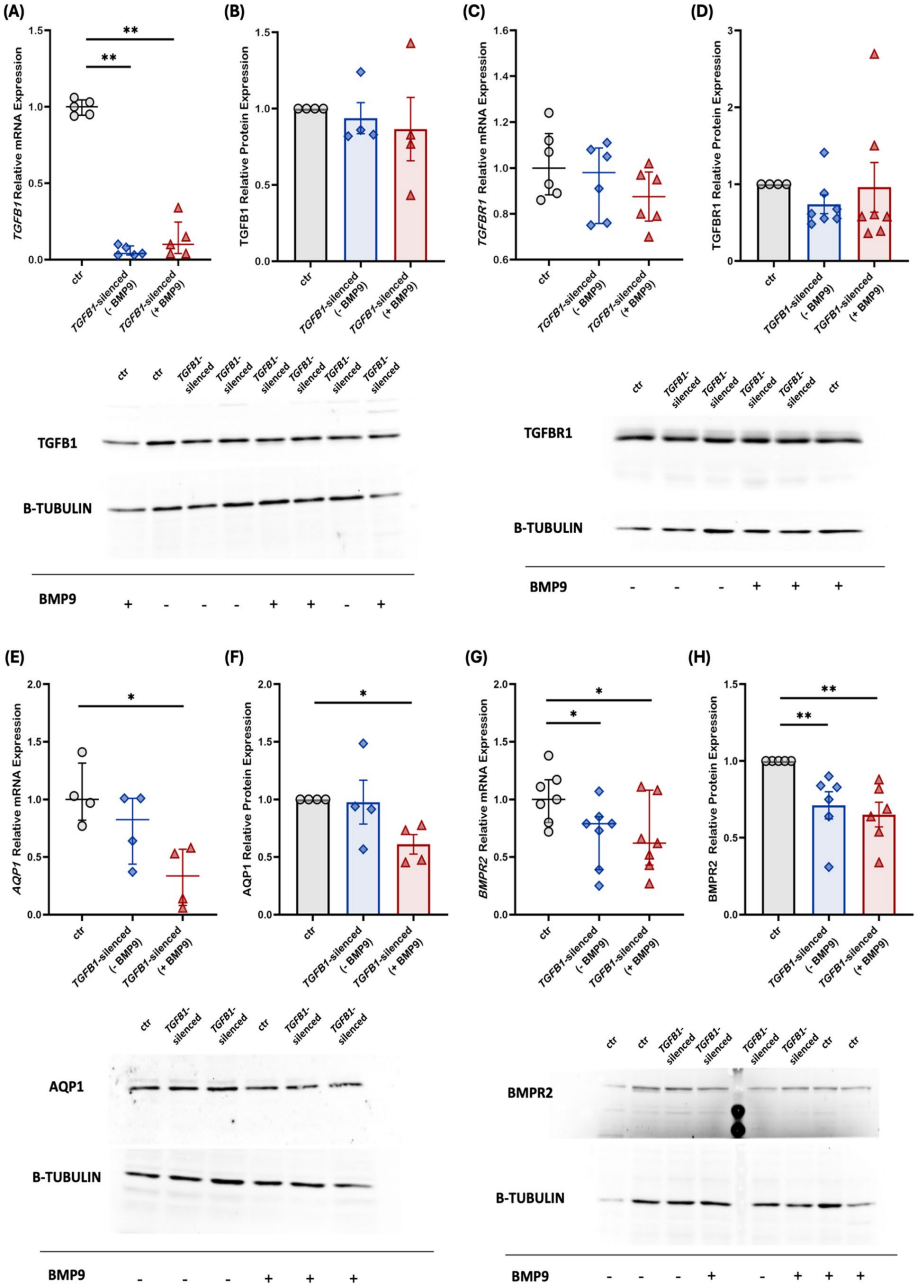
Effects of silencing the *TGFB1* gene and exogenous administration of BMP9 on BMP/TGF‐β signaling molecules and AQP1 in human pulmonary microvascular endothelial cells. HPMECs were silenced for the *TGFB1* gene and the relative mRNA and protein expression of TGFB1 (A, B), TGFBR1 (C, D), AQP1 (E, F), and BMPR2 (G, H) were estimated before and after the exogenous administration of BMP9. Relative mRNA expression is shown in dot plots (dots, individual values; line in the middle, median values; lower and upper lines, 25th and 75th percentiles) (A, *n* = 5; C, *n* = 6; E, *n* = 4; G, *n* = 7). Protein expression was analyzed by SDS‐PAGE and immunoblotting, and relative expression was estimated by densitometry using β‐tubulin or Actin as a loading control. Relative protein expression is shown with bar plots (mean ± SEM; dots, individual values) (B, *n* = 4; D, *n* = 7; F, *n* = 4; H, *n* = 6). Representative expression of TGFB1 (B), TGFBR1 (D), AQP1 (F), and BMPR2 (H) (upper panels) and β‐tubulin (lower panels) proteins in the *TGFB1*‐silenced HPMEC homogenates. Specificity (siRNA negative control), efficiency (*TGFB1* siRNA), and the effect of BMP9 exogenous administration on the non‐transfected controls were tested each time to ensure consistency and reproducibility across the independent experiments. Statistical analysis was performed using the Mann–Whitney test. *, *p* < 0.05; **, *p* < 0.01 compared to the non‐transfected control HPMECs.

In a prior study, our group demonstrated the effect of exogenous BMP9 administration on the dysregulated BMP/TGF‐β signaling pathway of HPMECs silenced for the *BMPR2* gene (Lotsios et al. 2024). Considering these results, we decided to explore the effects of silencing the *TGFB1* gene and the administration of BMP9 on key molecules of the BMP/TGF‐β signaling pathway. A universal scrambled negative control siRNA duplex (siRNA controls) was utilized for transfection specificity testing. Transfection of HPMECs with the *TGFB1*‐specific siRNA decreased *TGFB1* mRNA expression significantly in comparison to the non‐transfected control cells (0.04 [0.03–0.09] vs. 1.00 [0.95–1.05], respectively, *p* < 0.01; Figure 2A), while administration of exogenous BMP9 failed to impact *TGFB1* mRNA levels in transfected cells, when compared against non‐transfected control cells (0.10 [0.04–0.25], *p* < 0.01; Figure 2A). It should be noted that the siRNA control group also exhibited decreased expression levels compared to the non‐transfected control group, however higher than those of the transfected cells (0.56 [0.28–0.67], *p* < 0.01 vs. non‐transfected control cells and *p* < 0.01 vs. *TGFB1*‐silenced cells). In contrast to the mRNA expression, TGFB1 protein levels remained unaltered following *TGFB1 silencing* (0.94 ± 0.10, *p* > 0.05; Figure 2B) and after BMP9 treatment (0.87 ± 0.21, *p* > 0.05; Figure 2B) compared to the non‐transfected control cells. Considering the unchanged protein levels of TGFB1 post‐transfection, we proceeded to examine the principal downstream effectors of the TGF‐β family signaling, namely SMAD2, SMAD3, and SMAD4. In *TGFB1*‐silenced cells, *SMAD2* mRNA expression decreased compared to the non‐transfected control cells (0.38 [0.33–0.38] vs. 1.00 [0.89–1.06], respectively, *p* < 0.001; Figure S2A). Similarly, p‐SMAD2 protein levels presented a decrease in the *TGFB1*‐silenced cells when compared against the non‐transfected control cells (0.48 ± 0.12, *p* < 0.05; Figure S2B). Additionally, *SMAD3* mRNA levels were decreased in the *TGFB1*‐silenced cells compared to the non‐transfected control cells (0.54 [0.49–0.57] vs. 1.00 [0.93–1.14], respectively, *p* < 0.01; Figure S2C). Finally, *SMAD4* mRNA levels decreased in the *TGFB1*‐silenced cells compared to the non‐transfected control cells (0.55 [0.52–0.58] vs. 1.00 [0.92–1.01], respectively, *p* < 0.001; Figure S2D).

*TGFB1* gene silencing did not impact *TGFBR1* mRNA expression, when compared to the non‐transfected control cells (0.98 [0.76–1.09] vs. 1.00 [0.88–1.15], respectively, *p* > 0.05; Figure 2C). Furthermore, *TGFBR1* mRNA levels in the *TGFB1*‐silenced cells remained unaltered following BMP9 administration, in comparison to the non‐transfected control cells (0.88 [0.77–0.98], *p* > 0.05; Figure 2C). Similarly, no changes were discovered in TGFBR1 protein levels in the *TGFB1*‐silenced cells either pre‐ or post‐BMP9 treatment (0.74 ± 0.12, *p* > 0.05; Figure 2D and 0.96 ± 0.32, *p* > 0.05; Figure 2D, respectively) compared to the non‐transfected control cells.

Silencing the *TGFB1* gene did not affect *AQP1* mRNA levels compared to the non‐transfected control cells (0.83 [0.44–1.01] vs. 1.00 [0.82–1.32], respectively, *p* > 0.05; Figure 2E), BMP9 administration decreased *AQP1* mRNA expression significantly in the *TGFB1*‐silenced cells, compared to the non‐transfected control cells (0.34 [0.08–0.57], *p* < 0.05; Figure 2E). In the *TGFB1*‐silenced cells, AQP1 protein levels remained stable (0.98 ± 0.19, *p* > 0.05; Figure 2F). Exogenous administration of BMP9 resulted in a significant decrease in AQP1 protein compared to the non‐transfected control cells (0.61 ± 0.09, *p* < 0.05; Figure 2F).

HPMECs silenced for the *TGFB1* gene, demonstrated a significant decrease in *BMPR2* mRNA levels compared to the non‐transfected control cells (0.79 [0.39–0.85] vs. 1.00 [0.80–1.17], respectively, *p* < 0.05; Figure 2G). Compared to the non‐transfected control cells, treatment of the *TGFB1*‐silenced cells with exogenous BMP9 did not restore the reduced *BMPR2* mRNA levels (0.62 [0.43–1.08], *p* < 0.05; Figure 2G). BMPR2 protein levels followed a similar pattern to that of the mRNA. Initially, the *TGFB1*‐silenced cells demonstrated significantly lower levels of the BMPR2 protein (0.71 ± 0.09, *p* < 0.01; Figure 2H) compared to the non‐transfected control cells. BMPR2 protein levels remained low post‐exogenous administration of BMP9 (0.65 ± 0.08, *p* < 0.01; Figure 2H).

**FIGURE 3:**
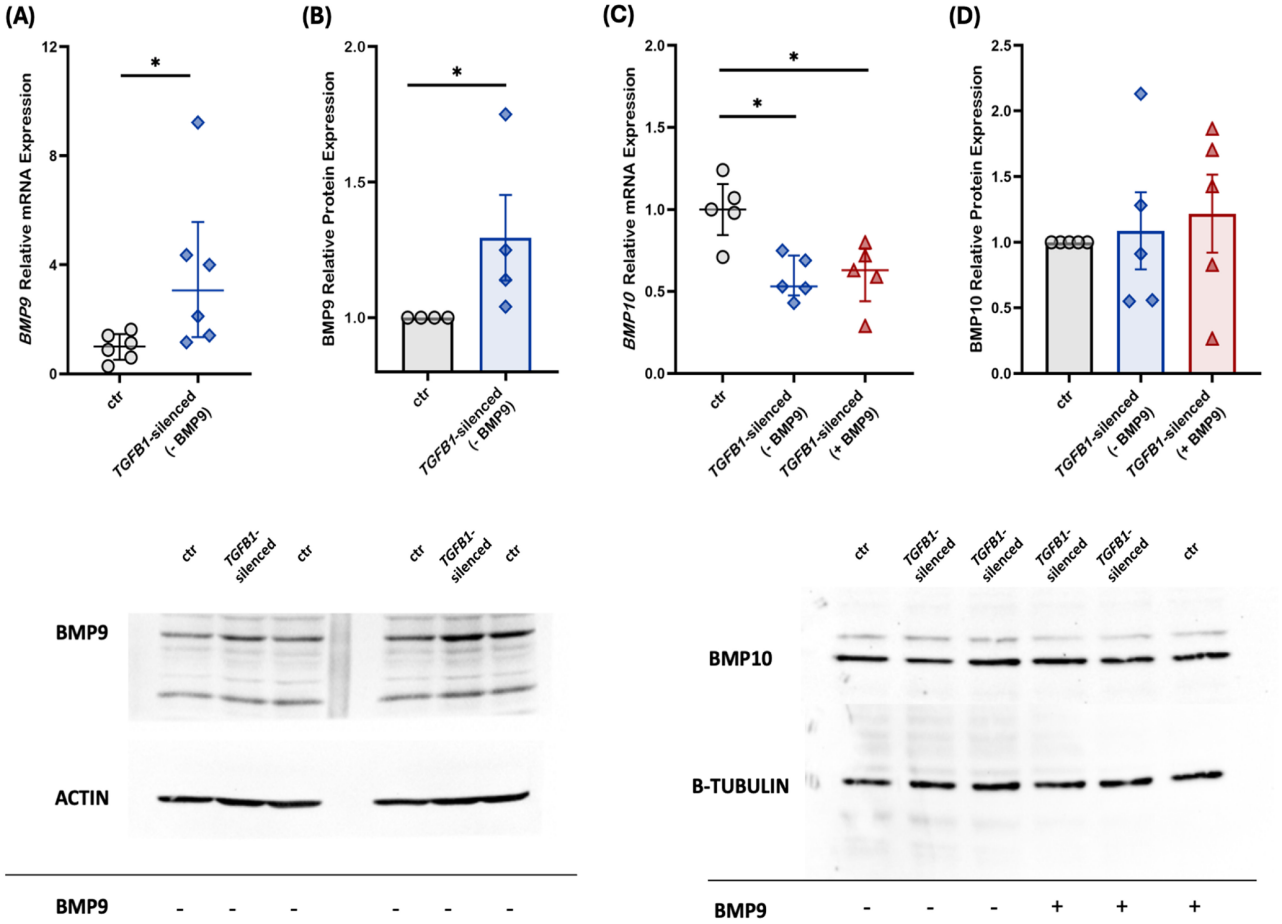
Effects of silencing the *TGFB1* gene and exogenous administration of BMP9 on BMP/TGF‐β ligands in human pulmonary microvascular endothelial cells. HPMECs were silenced for the *TGFB1* gene, and the relative mRNA and protein expression of BMP9 (A, B) and BMP10 (C, D) were estimated. BMP10 expression was estimated before and after the exogenous administration of BMP9. Relative mRNA expression is shown in dot plots (dots, individual values; line in the middle, median values; lower and upper lines, 25th and 75th percentiles) (A, *n* = 6; C, *n* = 5). Protein expression was analyzed by SDS‐PAGE and immunoblotting, and relative expression was estimated by densitometry using β‐tubulin or Actin as a loading control. Relative protein expression is shown with bar plots (mean ± SEM; dots, individual values) (B, *n* = 4; D, *n* = 5). Representative expression of BMP9 (B), and BMP10 (D) (upper panels) and β‐tubulin/Actin (lower panels) proteins in the *TGFB1*‐silenced HPMEC homogenates. Specificity (siRNA negative control), efficiency (*TGFB1* siRNA), and the effect of BMP9 exogenous administration on the non‐transfected controls were tested each time to ensure consistency and reproducibility across the independent experiments. Statistical analysis was performed using the Mann–Whitney test. *, *p* < 0.05 compared to the non‐transfected control HPMECs.

We then proceeded to evaluate the expression of BMP9 and BMP10, the main ligands of BMPR2, following silencing of the *TGFB1* gene. *BMP9* mRNA levels of the *TGFB1*‐silenced cells increased nearly 3‐fold compared to the non‐transfected control cells (3.06 [1.35–5.58] vs. 1.01 [0.52–1.46], respectively, *p* < 0.05; Figure 3A), while BMP9 protein demonstrated an increase equivalent to 29% in the *TGFB1*‐silenced cells (1.29 ± 0.16, *p* < 0.05; Figure 3B). On the other hand, *BMP10* mRNA levels were significantly decreased in the *TGFB1*‐silenced cells compared to the non‐transfected control cells (0.53 [0.48–0.72] vs. 1.00 [0.85–1.16], respectively, *p* < 0.05; Figure 3C). Exogenous BMP9 administration did not demonstrate any effect on *BMP10* mRNA levels (0.63 [0.44–0.76], *p* < 0.05, compared to the non‐transfected control cells; Figure 3C). In contrast to mRNA expression, BMP10 protein levels in the *TGFB1*‐silenced cells remained comparable to those of the non‐transfected control cells pre‐ and post‐exogenous BMP9 administration (1.09 ± 0.29, *p* > 0.05 and 1.22 ± 0.30, *p* > 0.05, respectively; Figure 3D).

## 4 Discussion

In this study, our goal was to delve deeper into the complex interactions between AQP1 and key molecules involved in the BMP/TGF‐β pathways and shed light on the effect of exogenous BMP9 treatment in HPMECs. We demonstrated that silencing of *AQP1* downregulated the mRNA expression of *BMPR2*, *TGFB1*, and *TGFBR1*, and BMPR2 protein levels. On the other hand, *TGFB1* silencing did not affect TGFBR1 and AQP1 expression, while it decreased BMPR2 mRNA and protein levels, and *BMP10* mRNA expression. Interestingly, *TGFB1*‐silencing increased the mRNA and protein levels of BMP9, a main ligand of BMPR2. BMP9 treatment failed to restore the reduced AQP1 and BMPR2 levels in *AQP1*‐silenced HPMECs, while it was successful in restoring *TGFB1* mRNA to control levels. In contrast, in *TGFB1*‐silenced HPMECs, BMP9 treatment did not restore the reduced BMPR2 levels, though it led to reduced AQP1 levels.

As mentioned above, endothelial dysfunction is a hallmark in PAH pathophysiology. ECs are characterized by high morphological and functional heterogeneity across vascular beds (Kalucka et al. 2020). They aid lung vascular remodeling either directly by increasing cell proliferation and inducing antiapoptotic pathways, or indirectly by releasing growth factors that stimulate the proliferation and migration of pulmonary artery smooth muscle cells (PASMCs) (Masri et al. 2007; Guignabert et al. 2009). Recently, Zhang et al. proposed a new mechanism regarding small vessel muscularization in pulmonary hypertension, suggesting that apoptosis and proliferation occur concurrently; however in different EC subsets (Zhang et al. 2024).

In this study, we performed our experiments on HPMECs, since our group has previously explored the interplay between AQP1, BMPR2, and TGFB1 in this cell type. In 2021, we were the first to report that in *AQP1*‐silenced HPMECs, *BMPR2* mRNA expression levels were reduced, suggesting a possible crosstalk between the two pathways (Vassiliou et al. 2021). In the same study, we also observed reduced *TGFB1* mRNA levels in *AQP1*‐silenced HPMECs (Vassiliou et al. 2021). We have also demonstrated a dysfunctional AQP1 in terms of reduced levels and permeability function in *BMPR2*‐silenced HPMECs (Vassiliou et al. 2020). Aiming to broaden our understanding of their interaction patterns, we further explored the effect of AQP1 silencing on BMPR2, and the key receptor‐ligand duo, TGFB1 and TGFBR1. Subsequently, we examined the expression patterns of BMP9, BMP10, AQP1, BMPR2, TGFB1, and TGFBR1 in *TGFB1*‐silenced HPMECs.

Recently, a whole genome sequencing analysis of over 1000 patients with idiopathic and heritable forms of PAH found rare and likely causal heterozygous variants in the *AQP1* gene (Gräf et al. 2018). Apart from fluid hemostasis, AQP1 plays a key role in endothelial cell migration and angiogenesis (Saadoun et al. 2005). Studies on various hypoxia‐induced pulmonary hypertension (HPH) rodent models have demonstrated the role of AQP1 in the proliferation and migration of PASMCs. More specifically, AQP1 expression was induced in response to hypoxia, while depletion of the *AQP1* gene reduced hypoxia‐induced proliferation, PASMCs' migratory potential, and induced apoptosis, suggesting that AQP1 could be a potential target for the treatment of HPH (Leggett et al. 2012; Schuoler et al. 2017; Liu et al. 2019). Similar findings were also reported in pulmonary microvascular endothelial cells (MVECs) in a Sugen/Hypoxia rat model of severe PH, a model that better replicated the pathophysiological features of human PAH (Yun et al. 2021). Herein, besides reconfirming that silencing of the *AQP1* gene results in reduced *BMPR2* mRNA (Vassiliou et al. 2021; Lotsios et al. 2024), we were able to demonstrate that it also reduces its protein levels. These findings strengthen the evidence for a previously unknown interdependent relationship between AQP1 and BMPR2, at both the mRNA and protein levels in HPMECs. We also observed that silencing of the *AQP1* gene resulted in reduced *TGFBR1* and *TGFB1* mRNA expression, while it tended to reduce TGFBR1 protein levels. These results combined with our group's previous findings that silencing of the *BMPR2* gene negatively affects TGFB1 expression (Vassiliou et al. 2020; Lotsios et al. 2024), suggest that silencing of both the *AQP1* and *BMPR2* genes contributes to the dysregulated expression of *TGFB1* mRNA.

Until now, most studies have focused on the downstream signaling of BMPR2; however, there is a need to include the role of signaling through TGF‐β in PAH. Although BMP ligands and their receptors play an important role in the progression of the disease and could serve as therapeutic targets (Long et al. 2015), agents that effectively reduce TGF‐β1 activity and selective ligand traps, open up new therapeutic approaches (Ogo et al. 2013; Lu et al. 2015; Yung et al. 2016; Bellaye et al. 2018; Zabini et al. 2018). Increased TGF‐β1 levels have been found systemically and locally in both PAH patients and animal models (Rol et al. 2018). Furthermore, studies have demonstrated that TGF‐β signaling can directly inhibit the BMP/SMAD pathway in PASMCs and that TGF‐β ligands can function as antagonists by competing for type II receptor binding (Upton et al. 2013; Aykul and Martinez‐Hackert 2016). It has therefore been suggested that inhibition of the TGF‐β signaling pathways may constitute a novel therapeutic approach in PAH.

Herein, we silenced HPMECs for the *TGFB1* gene and explored the modulated expression of BMP/TGF‐β‐related signaling molecules. Silencing of the *TGFB1* gene negatively affected BMPR2 mRNA and protein expression, while it didn't affect AQP1 and TGFBR1 expression. These findings suggest that TGFB1 could possibly participate in the regulation of BMPR2, without possessing a significant regulatory effect on AQP1. Regarding the BMPR2 ligands, BMP9 expression levels demonstrated a significant increase in *TGFB1*‐silenced cells, which was similar to the expression pattern described previously in HPMECs silenced for the *AQP1* and *BMPR2* genes. Following silencing of the *TGFB1* gene, *BMP10* mRNA expression decreased, in contrast to its increased mRNA expression observed in *AQP1*‐ and *BMPR2*‐silenced HPMECs (Lotsios et al. 2024).

It is well established that imbalanced BMP signaling is a causative factor of PAH progression. BMP9 is a growth factor belonging to the TGF‐β superfamily. It is one of the main ligands of BMPR2 and a known circulating vascular‐inducing factor. Depending on the dose and vascular context, BMP9 exhibits both pro‐ and anti‐angiogenic roles, and these functions may further differ in quiescence and dysregulated vascular beds (García de Vinuesa et al. 2016). BMP9 has been suggested as a possible therapeutic strategy for PAH based on the hypothesis that supplementation of exogenous BMP9 could enhance endothelial BMPR2 signaling and reverse PAH in in vivo models (Long et al. 2015). However, findings from in vivo and in vitro PAH models are thus far conflicting (Upton et al. 2009; Nikolic et al. 2019; Tu et al. 2019; Theilmann et al. 2020; Wang et al. 2021; Bouvard et al. 2022). These conflicting results could suggest that its effects may vary at different stages of PAH; it might have a beneficial role in early disease stages, while its role seems to be more complicated in later stages of the disease (Li and Quigley 2024).

Previously published data from our group suggested that in HPMECs silenced for the *BMPR2* gene, the effect of the exogenous administration of BMP9 on molecules participating in the BMP/TGF‐β signaling pathways seems to depend on *BMPR2* mRNA expression levels (Lotsios et al. 2024). In the present study, we observed that the exogenous administration of BMP9 in *AQP1*‐silenced HPMECs did not revert the decreased mRNA and protein levels of AQP1, BMPR2, and TGFBR1, while BMP9 treatment restored *TGFB1* mRNA levels. It is plausible that BMP9's effect on AQP1 is likely a result of the complex interplay between BMPR2 expression levels and an unknown molecular mechanism regulating its expression. On the other hand, in the *TGFB1*‐silenced HPMECs, exogenous administration of BMP9 reduced the pre‐BMP9 treatment AQP1 mRNA and protein levels, while it did not reverse the reduced BMPR2 levels. Hence, BMP9 does not restore BMPR2 expression when either *AQP1* or *TGFB1* genes are silenced in HPMECs, thus raising the possibility that its effect on BMPR2 is independent of the molecular mechanism by which BMPR2 expression is downregulated. It is possible that the effects of BMP9 could depend on the expression levels of the genes silenced, and that these effects apply in the cell type studied in our work.

Interestingly, Szulcek et al. examined the effect of BMP9 in HPMECs isolated from pleura‐free peripheral lung tissues of PAH patients (Szulcek et al. 2020). They found that HPMECs from PAH patients responded to BMP9 stimulation, exhibiting an 8‐fold increase in BMPR2 levels, differing from the response observed in PAECs and endothelial colony‐forming cells (ECFCs), thus suggesting that the lung tissue microenvironment could affect activation and outcome of BMP‐dependent signaling pathways in PAH. More specifically, in PAH MVECs, BMP9 activated persistent EndMT signaling, leading to the loss of endothelial‐specific markers, gaining of mesenchymal characteristics, and decreased barrier integrity (Szulcek et al. 2020).

As stated above, the focus of our study was to explore the effects of silencing the *AQP1* and *TGFB1* genes in HPMECs on the BMP/TGF‐β signaling pathway. The limitations of our study must be acknowledged. While AQP1 mRNA and protein expression were decreased post‐transfection, the reduction of TGFB1 expression levels was evident only at the mRNA level in our set of experiments (cells were harvested at 48 h due to increased cytotoxicity). Knowing that TGFB1 is secreted in an inactive/latent form bound to TGF binding proteins in the extracellular matrix, we hypothesize that its protein levels could persist even after mRNA expression is silenced. However, it is also worth mentioning that we demonstrated that the mRNA levels of *SMAD2/3* and *SMAD4*, and the protein levels of p‐SMAD2 were decreased, indicating the disruption of the TGF‐β/SMAD2/3 signaling pathway. Furthermore, among the limitations of this study is that our experiments mainly focused on the genetic and protein expression of molecules known to interact with the BMP/TGF‐β signaling pathway and we did not assess the proliferative and/or migratory behavior of the cells. In this study, we performed our experiments in a single in vitro cell model (HPMECs), since our group has previously explored the interplay between AQP1, BMPR2, and TGFB1 in this cell type. However, additional studies or co‐culture studies performed on established cell models, animal PAH models, or cell samples extracted from PAH patients, including induced pluripotent stem cells (iPSCs), could be beneficial. While our study provides novel insights into how BMP9 treatment affects the BMP/TGF‐β signaling pathway, it should be stated that these results reflect specifically the ECs of the pulmonary microvasculature. Further studies exploring the effect of BMP9 on other cell types known to participate in the pathophysiology of PAH such as PASMCs, could demonstrate a differential effect on the genetic level. Importantly, additional clinical studies should be employed to further investigate the therapeutic potential of BMP9. It must be noted that one of the key aspects of our study is the fact that it provides novel insight into the established interaction of AQP1 and BMPR2. To the best of our knowledge, the present study is the first to demonstrate the effect of BMP9 on the complex interactions between AQP1 and key molecules of the BMP/TGF‐β signaling pathway, at a molecular and protein level in HPMECs in the setting of either hindered *AQP1* or *TGFB1* gene expression.

## 5 Conclusions

Our findings suggest that in HPMECs, silencing of either the *AQP1* or *TGFB1* gene, along with BMP9 administration alters the expression of key molecules in the BMP/TGF‐β signaling pathway. Disruptions in this complex pathway may directly impact endothelial function, though this finding seems to be cell‐ and context‐dependent. While our results further support the proposed interplay between AQP1 and BMPR2 in the canonical BMP/TGF‐β signaling pathway, the specifics of this interaction are unknown. Additionally, though the function of BMP9 has yet to be fully understood in different PAH models and stages of the disease, its effect seems to differ depending on the genetic background and/or the condition of the vascular bed. Further studies across different cell types known to participate in PAH development and progression could unravel the complex interactions, leading to a better understanding of BMP9's therapeutic potential.

## Author Contributions

**Stylianos E. Orfanos** and **Alice G. Vassiliou:** conceptualization. **Chrysi Keskinidou**, **Nikolaos S. Lotsios**, **Kostas A. Papavassiliou**, and **Alice G. Vassiliou:** methodology. **Athanasios G. Papavassiliou**, **Ioanna Dimopoulou**, **Anastasia Kotanidou**, and **Alice G. Vassiliou:** validation. **Chrysi Keskinidou**, **Nikolaos S. Lotsios**, **Kostas A. Papavassiliou**, and **Alice G. Vassiliou:** formal analysis. **Chrysi Keskinidou**, **Nikolaos S. Lotsios**, and **Alice G. Vassiliou:** investigation. **Stylianos E. Orfanos** and **Alice G. Vassiliou:** resources. **Chrysi Keskinidou** and **Nikolaos S. Lotsios:** data curation. **Chrysi Keskinidou**, **Nikolaos S. Lotsios**, and **Kostas A. Papavassiliou:** writing – original draft preparation. **Athanasios G. Papavassiliou**, **Ioanna Dimopoulou**, **Anastasia Kotanidou**, **David Langleben**, **Stylianos E. Orfanos**, and **Alice G. Vassiliou:** writing – review and editing. **David Langleben**, **Stylianos E. Orfanos**, and **Alice G. Vassiliou:** visualization. **David Langleben**, **Stylianos E. Orfanos**, and **Alice G. Vassiliou:** supervision. **Athanasios G. Papavassiliou**, **Ioanna Dimopoulou**, **Anastasia Kotanidou**, **David Langleben**, **Stylianos E. Orfanos**, and **Alice G. Vassiliou:** project administration. All authors have read and agreed to the published version of the manuscript.

## Conflicts of Interest

The authors declare no conflicts of interest.

## Supporting information


**Figure S1:** cph470066‐sup‐0001‐FigureS1.pdf.

**Figure S2:** cph470066‐sup‐0002‐FigureS2.pdf.

## Data Availability

The data supporting this study's findings are available from the corresponding author upon reasonable request.

## References

[cph470066-bib-0001] Adu‐Amankwaah, J. , Q. You , X. Liu , et al. 2025. “Pulmonary Hypertension: Molecular Mechanisms and Clinical Studies.” MedComm 6, no. 3: e70134. 40066229 10.1002/mco2.70134 PMC11892029

[cph470066-bib-0002] Austin, E. D. , M. A. Aldred , M. Alotaibi , et al. 2024. “Genetics and Precision Genomics Approaches to Pulmonary Hypertension.” European Respiratory Journal 64, no. 4: 2401370. 39209481 10.1183/13993003.01370-2024 PMC11525347

[cph470066-bib-0003] Aykul, S. , and E. Martinez‐Hackert . 2016. “Transforming Growth Factor‐β Family Ligands Can Function as Antagonists by Competing for Type ii Receptor Binding.” Journal of Biological Chemistry 291, no. 20: 10792–10804. 26961869 10.1074/jbc.M115.713487 PMC4865925

[cph470066-bib-0004] Bellaye, P. S. , T. Yanagihara , E. Granton , et al. 2018. “Macitentan Reduces Progression of Tgf‐β1‐Induced Pulmonary Fibrosis and Pulmonary Hypertension.” European Respiratory Journal 52, no. 2: 1701857. 29976656 10.1183/13993003.01857-2017

[cph470066-bib-0005] Bousseau, S. , R. Sobrano Fais , S. Gu , A. Frump , and T. Lahm . 2023. “Pathophysiology and New Advances in Pulmonary Hypertension.” BMJ Medicine 2, no. 1: e000137. 37051026 10.1136/bmjmed-2022-000137 PMC10083754

[cph470066-bib-0006] Bouvard, C. , L. Tu , M. Rossi , et al. 2022. “Different Cardiovascular and Pulmonary Phenotypes for Single‐ and Double‐Knock‐Out Mice Deficient in bmp9 and bmp10.” Cardiovascular Research 118, no. 7: 1805–1820. 34086873 10.1093/cvr/cvab187 PMC9215199

[cph470066-bib-0007] Cober, N. D. , M. M. VandenBroek , M. L. Ormiston , and D. J. Stewart . 2022. “Evolving Concepts in Endothelial Pathobiology of Pulmonary Arterial Hypertension.” Hypertension 79, no. 8: 1580–1590. 35582968 10.1161/HYPERTENSIONAHA.122.18261

[cph470066-bib-0008] Evans, C. E. , N. D. Cober , Z. Dai , D. J. Stewart , and Y. Y. Zhao . 2021. “Endothelial Cells in the Pathogenesis of Pulmonary Arterial Hypertension.” European Respiratory Journal 58, no. 3: 2003957. 33509961 10.1183/13993003.03957-2020 PMC8316496

[cph470066-bib-0009] Evans, J. D. , B. Girerd , D. Montani , et al. 2016. “Bmpr2 Mutations and Survival in Pulmonary Arterial Hypertension: An Individual Participant Data Meta‐Analysis.” Lancet Respiratory Medicine 4, no. 2: 129–137. 26795434 10.1016/S2213-2600(15)00544-5 PMC4737700

[cph470066-bib-0010] García de Vinuesa, A. , S. Abdelilah‐Seyfried , P. Knaus , A. Zwijsen , and S. Bailly . 2016. “Bmp Signaling in Vascular Biology and Dysfunction.” Cytokine & Growth Factor Reviews 27: 65–79. 26823333 10.1016/j.cytogfr.2015.12.005

[cph470066-bib-0011] Gorelova, A. , M. Berman , and I. Al Ghouleh . 2021. “Endothelial‐To‐Mesenchymal Transition in Pulmonary Arterial Hypertension.” Antioxidants & Redox Signaling 34, no. 12: 891–914. 32746619 10.1089/ars.2020.8169 PMC8035923

[cph470066-bib-0012] Gräf, S. , M. Haimel , M. Bleda , et al. 2018. “Identification of Rare Sequence Variation Underlying Heritable Pulmonary Arterial Hypertension.” Nature Communications 9, no. 1: 1416. 10.1038/s41467-018-03672-4 PMC5897357 29650961

[cph470066-bib-0013] Guignabert, C. , C. M. Alvira , T. P. Alastalo , et al. 2009. “Tie2‐Mediated Loss of Peroxisome Proliferator‐Activated Receptor‐Gamma in Mice Causes Pdgf Receptor‐Beta‐Dependent Pulmonary Arterial Muscularization.” American Journal of Physiology Lung Cellular and Molecular Physiology 297, no. 6: L1082–L1090. 19801450 10.1152/ajplung.00199.2009 PMC2793182

[cph470066-bib-0014] Heldin, C. H. , and A. Moustakas . 2012. “Role of Smads in tgfβ Signaling.” Cell and Tissue Research 347, no. 1: 21–36. 21643690 10.1007/s00441-011-1190-x

[cph470066-bib-0015] Huertas, A. , C. Guignabert , J. A. Barberà , et al. 2018. “Pulmonary Vascular Endothelium: The Orchestra Conductor in Respiratory Diseases: Highlights From Basic Research to Therapy.” European Respiratory Journal 51, no. 4: 1700745. 29545281 10.1183/13993003.00745-2017

[cph470066-bib-0016] Humbert, M. , C. Guignabert , S. Bonnet , et al. 2019. “Pathology and Pathobiology of Pulmonary Hypertension: State of the Art and Research Perspectives.” European Respiratory Journal 53, no. 1: 1801887. 30545970 10.1183/13993003.01887-2018 PMC6351340

[cph470066-bib-0017] Kalucka, J. , L. de Rooij , J. Goveia , et al. 2020. “Single‐Cell Transcriptome Atlas of Murine Endothelial Cells.” Cell 180, no. 4: 764–779. 32059779 10.1016/j.cell.2020.01.015

[cph470066-bib-0018] Katta, S. , S. Vadapalli , B. K. Sastry , and P. Nallari . 2008. “T‐Plasminogen Activator Inhibitor‐1 Polymorphism in Idiopathic Pulmonary Arterial Hypertension.” Indian Journal of Human Genetics 14, no. 2: 37–40. 20300292 10.4103/0971-6866.44103 PMC2840792

[cph470066-bib-0019] Krump‐Konvalinkova, V. , F. Bittinger , R. E. Unger , K. Peters , H. A. Lehr , and C. J. Kirkpatrick . 2001. “Generation of Human Pulmonary Microvascular Endothelial Cell Lines.” Laboratory Investigation 81, no. 12: 1717–1727. 11742042 10.1038/labinvest.3780385

[cph470066-bib-0020] Laemmli, U. K. 1970. “Cleavage of Structural Proteins During the Assembly of the Head of Bacteriophage t4.” Nature 227, no. 5259: 680–685. 5432063 10.1038/227680a0

[cph470066-bib-0021] Leggett, K. , J. Maylor , C. Undem , et al. 2012. “Hypoxia‐Induced Migration in Pulmonary Arterial Smooth Muscle Cells Requires Calcium‐Dependent Upregulation of Aquaporin 1.” American Journal of Physiology Lung Cellular and Molecular Physiology 303, no. 4: L343–L353. 22683574 10.1152/ajplung.00130.2012 PMC3423828

[cph470066-bib-0022] Li, W. , and K. Quigley . 2024. “Bone Morphogenetic Protein Signalling in Pulmonary Arterial Hypertension: Revisiting the Bmprii Connection.” Biochemical Society Transactions 52, no. 3: 1515–1528. 38716930 10.1042/BST20231547 PMC11346422

[cph470066-bib-0023] Liu, M. , Q. Liu , Y. Pei , et al. 2019. “Aqp‐1 Gene Knockout Attenuates Hypoxic Pulmonary Hypertension of Mice.” Arteriosclerosis, Thrombosis, and Vascular Biology 39, no. 1: 48–62. 30580569 10.1161/ATVBAHA.118.311714

[cph470066-bib-0024] Livak, K. J. , and T. D. Schmittgen . 2001. “Analysis of Relative Gene Expression Data Using Real‐Time Quantitative Pcr and the 2(−Delta Delta c(t)) Method.” Methods 25, no. 4: 402–408. 11846609 10.1006/meth.2001.1262

[cph470066-bib-0025] Lleu, P. L. , and G. Rebel . 1991. “Interference of Good's Buffers and Other Biological Buffers With Protein Determination.” Analytical Biochemistry 192, no. 1: 215–218. 2048724 10.1016/0003-2697(91)90210-k

[cph470066-bib-0026] Long, L. , M. L. Ormiston , X. Yang , et al. 2015. “Selective Enhancement of Endothelial Bmpr‐ii With bmp9 Reverses Pulmonary Arterial Hypertension.” Nature Medicine 21, no. 7: 777–785. 10.1038/nm.3877 PMC4496295 26076038

[cph470066-bib-0027] Lotsios, N. S. , C. Keskinidou , I. Dimopoulou , et al. 2024. “Effects of Modulating bmp9, bmpr2, and aqp1 on Bmp Signaling in Human Pulmonary Microvascular Endothelial Cells.” International Journal of Molecular Sciences 25: 8043. 39125626 10.3390/ijms25158043 PMC11311989

[cph470066-bib-0028] Lotsios, N. S. , C. Keskinidou , I. Dimopoulou , A. Kotanidou , S. E. Orfanos , and A. G. Vassiliou . 2023. “Aquaporin Expression and Regulation in Clinical and Experimental Sepsis.” International Journal of Molecular Sciences 25, no. 1: 487. 38203657 10.3390/ijms25010487 PMC10778766

[cph470066-bib-0029] Lu, A. , C. Zuo , Y. He , et al. 2015. “Ep3 Receptor Deficiency Attenuates Pulmonary Hypertension Through Suppression of Rho/Tgf‐β1 Signaling.” Journal of Clinical Investigation 125, no. 3: 1228–1242. 25664856 10.1172/JCI77656 PMC4362262

[cph470066-bib-0030] Marinho, Y. , E. S. Villarreal , O. Loya , and S. D. Oliveira . 2024. “Mechanisms of Lung Endothelial Cell Injury and Survival in Pulmonary Arterial Hypertension.” American Journal of Physiology Lung Cellular and Molecular Physiology 327, no. 6: L972–l983. 39406383 10.1152/ajplung.00208.2024 PMC11684956

[cph470066-bib-0031] Masri, F. A. , W. Xu , S. A. Comhair , et al. 2007. “Hyperproliferative Apoptosis‐Resistant Endothelial Cells in Idiopathic Pulmonary Arterial Hypertension.” American Journal of Physiology Lung Cellular and Molecular Physiology 293, no. 3: L548–L554. 17526595 10.1152/ajplung.00428.2006

[cph470066-bib-0032] Meli, R. , C. Pirozzi , and A. Pelagalli . 2018. “New Perspectives on the Potential Role of Aquaporins (Aqps) in the Physiology of Inflammation.” Frontiers in Physiology 9: 101. 29503618 10.3389/fphys.2018.00101 PMC5820367

[cph470066-bib-0033] Morrell, N. W. , M. A. Aldred , W. K. Chung , et al. 2019. “Genetics and Genomics of Pulmonary Arterial Hypertension.” European Respiratory Journal 53, no. 1: 1801899. 30545973 10.1183/13993003.01899-2018 PMC6351337

[cph470066-bib-0034] Nikolic, I. , L. M. Yung , P. Yang , et al. 2019. “Bone Morphogenetic Protein 9 Is a Mechanistic Biomarker of Portopulmonary Hypertension.” American Journal of Respiratory and Critical Care Medicine 199, no. 7: 891–902. 30312106 10.1164/rccm.201807-1236OC PMC6444661

[cph470066-bib-0035] Ogo, T. , H. M. Chowdhury , J. Yang , et al. 2013. “Inhibition of Overactive Transforming Growth Factor‐β Signaling by Prostacyclin Analogs in Pulmonary Arterial Hypertension.” American Journal of Respiratory Cell and Molecular Biology 48, no. 6: 733–741. 23418342 10.1165/rcmb.2012-0049OC

[cph470066-bib-0036] Preston, I. R. , D. Lewis , and M. Gomberg‐Maitland . 2024. “Using Sotatercept in the Care of Patients With Pulmonary Arterial Hypertension.” Chest 166, no. 3: 604–611. 39004216 10.1016/j.chest.2024.06.3801

[cph470066-bib-0037] Rol, N. , K. B. Kurakula , C. Happé , H. J. Bogaard , and M. J. Goumans . 2018. “Tgf‐β and bmpr2 Signaling in Pah: Two Black Sheep in One Family.” International Journal of Molecular Sciences 19, no. 9: 2585. 30200294 10.3390/ijms19092585 PMC6164161

[cph470066-bib-0038] Saadoun, S. , M. C. Papadopoulos , M. Hara‐Chikuma , and A. S. Verkman . 2005. “Impairment of Angiogenesis and Cell Migration by Targeted Aquaporin‐1 Gene Disruption.” Nature 434, no. 7034: 786–792. 15815633 10.1038/nature03460

[cph470066-bib-0039] Schuoler, C. , T. J. Haider , C. Leuenberger , et al. 2017. “Aquaporin 1 Controls the Functional Phenotype of Pulmonary Smooth Muscle Cells in Hypoxia‐Induced Pulmonary Hypertension.” Basic Research in Cardiology 112, no. 3: 30. 28409279 10.1007/s00395-017-0620-7

[cph470066-bib-0040] Szulcek, R. , G. Sanchez‐Duffhues , N. Rol , et al. 2020. “Exacerbated Inflammatory Signaling Underlies Aberrant Response to bmp9 in Pulmonary Arterial Hypertension Lung Endothelial Cells.” Angiogenesis 23, no. 4: 699–714. 32813135 10.1007/s10456-020-09741-x PMC7524846

[cph470066-bib-0041] Theilmann, A. L. , L. G. Hawke , L. R. Hilton , et al. 2020. “Endothelial bmpr2 Loss Drives a Proliferative Response to Bmp (Bone Morphogenetic Protein) 9 via Prolonged Canonical Signaling.” Arteriosclerosis, Thrombosis, and Vascular Biology 40, no. 11: 2605–2618. 32998516 10.1161/ATVBAHA.119.313357 PMC7571847

[cph470066-bib-0042] Tu, L. , A. Desroches‐Castan , C. Mallet , et al. 2019. “Selective Bmp‐9 Inhibition Partially Protects Against Experimental Pulmonary Hypertension.” Circulation Research 124, no. 6: 846–855. 30636542 10.1161/CIRCRESAHA.118.313356

[cph470066-bib-0043] Upton, P. D. , R. J. Davies , T. Tajsic , and N. W. Morrell . 2013. “Transforming Growth Factor‐β(1) Represses Bone Morphogenetic Protein‐Mediated Smad Signaling in Pulmonary Artery Smooth Muscle Cells via smad3.” American Journal of Respiratory Cell and Molecular Biology 49, no. 6: 1135–1145. 23937428 10.1165/rcmb.2012-0470OC PMC3931109

[cph470066-bib-0044] Upton, P. D. , R. J. Davies , R. C. Trembath , and N. W. Morrell . 2009. “Bone Morphogenetic Protein (Bmp) and Activin Type ii Receptors Balance bmp9 Signals Mediated by Activin Receptor‐Like Kinase‐1 in Human Pulmonary Artery Endothelial Cells.” Journal of Biological Chemistry 284, no. 23: 15794–15804. 19366699 10.1074/jbc.M109.002881 PMC2708876

[cph470066-bib-0045] Vassiliou, A. G. , C. Keskinidou , A. Kotanidou , et al. 2020. “Knockdown of Bone Morphogenetic Protein Type ii Receptor Leads to Decreased Aquaporin 1 Expression and Function in Human Pulmonary Microvascular Endothelial Cells.” Canadian Journal of Physiology and Pharmacology 98, no. 11: 834–839. 32687728 10.1139/cjpp-2020-0185

[cph470066-bib-0046] Vassiliou, A. G. , C. Keskinidou , A. Kotanidou , et al. 2021. “Decreased Bone Morphogenetic Protein Type ii Receptor and Bmp‐Related Signalling Molecules' Expression in Aquaporin 1‐Silenced Human Pulmonary Microvascular Endothelial Cells.” Hellenic Journal of Cardiology 62, no. 1: 84–86. 32304816 10.1016/j.hjc.2020.04.003

[cph470066-bib-0047] Wang, L. , M. Rice , S. Swist , et al. 2021. “Bmp9 and bmp10 Act Directly on Vascular Smooth Muscle Cells for Generation and Maintenance of the Contractile State.” Circulation 143, no. 14: 1394–1410. 33334130 10.1161/CIRCULATIONAHA.120.047375

[cph470066-bib-0048] Welch, C. L. , M. A. Aldred , S. Balachandar , et al. 2023. “Defining the Clinical Validity of Genes Reported to Cause Pulmonary Arterial Hypertension.” Genetics in Medicine 25, no. 11: 100925. 37422716 10.1016/j.gim.2023.100925 PMC10766870

[cph470066-bib-0049] Yang, J. , X. Li , R. S. Al‐Lamki , et al. 2010. “Smad‐Dependent and Smad‐Independent Induction of id1 by Prostacyclin Analogues Inhibits Proliferation of Pulmonary Artery Smooth Muscle Cells In Vitro and In Vivo.” Circulation Research 107, no. 2: 252–262. 20522807 10.1161/CIRCRESAHA.109.209940

[cph470066-bib-0050] Yang, J. , X. Li , Y. Li , et al. 2013. “Id Proteins Are Critical Downstream Effectors of Bmp Signaling in Human Pulmonary Arterial Smooth Muscle Cells.” American Journal of Physiology Lung Cellular and Molecular Physiology 305, no. 4: L312–L321. 23771884 10.1152/ajplung.00054.2013 PMC3891012

[cph470066-bib-0051] Yun, X. , N. M. Philip , H. Jiang , et al. 2021. “Upregulation of Aquaporin 1 Mediates Increased Migration and Proliferation in Pulmonary Vascular Cells From the Rat su5416/Hypoxia Model of Pulmonary Hypertension.” Frontiers in Physiology 12: 763444. 34975522 10.3389/fphys.2021.763444 PMC8718640

[cph470066-bib-0052] Yung, L. M. , I. Nikolic , S. D. Paskin‐Flerlage , R. S. Pearsall , R. Kumar , and P. B. Yu . 2016. “A Selective Transforming Growth Factor‐β Ligand Trap Attenuates Pulmonary Hypertension.” American Journal of Respiratory and Critical Care Medicine 194, no. 9: 1140–1151. 27115515 10.1164/rccm.201510-1955OC PMC5114445

[cph470066-bib-0053] Zabini, D. , E. Granton , Y. Hu , et al. 2018. “Loss of smad3 Promotes Vascular Remodeling in Pulmonary Arterial Hypertension via Mrtf Disinhibition.” American Journal of Respiratory and Critical Care Medicine 197, no. 2: 244–260. 29095649 10.1164/rccm.201702-0386OC

[cph470066-bib-0054] Zhang, Q. , N. Yaoita , A. Tabuchi , et al. 2024. “Endothelial Heterogeneity in the Response to Autophagy Drives Small Vessel Muscularization in Pulmonary Hypertension.” Circulation 150, no. 6: 466–487. 38873770 10.1161/CIRCULATIONAHA.124.068726

