# Supplementary material for: Disrupting BMP/TGF‐β Signaling: Modulation of AQP1 and TGFB1 in Human Pulmonary Microvascular Endothelial Cells
Source: Compr Physiol. 2025 Oct 29;15(6):e70066. doi: 10.1002/cph4.70066 (PMC12570780; doi:10.1002/cph4.70066)
Supplement: Supplementary file 1 — Figure S1: cph470066‐sup‐0001‐FigureS1.pdf. [file CPH4-15-e70066-s002.pdf]

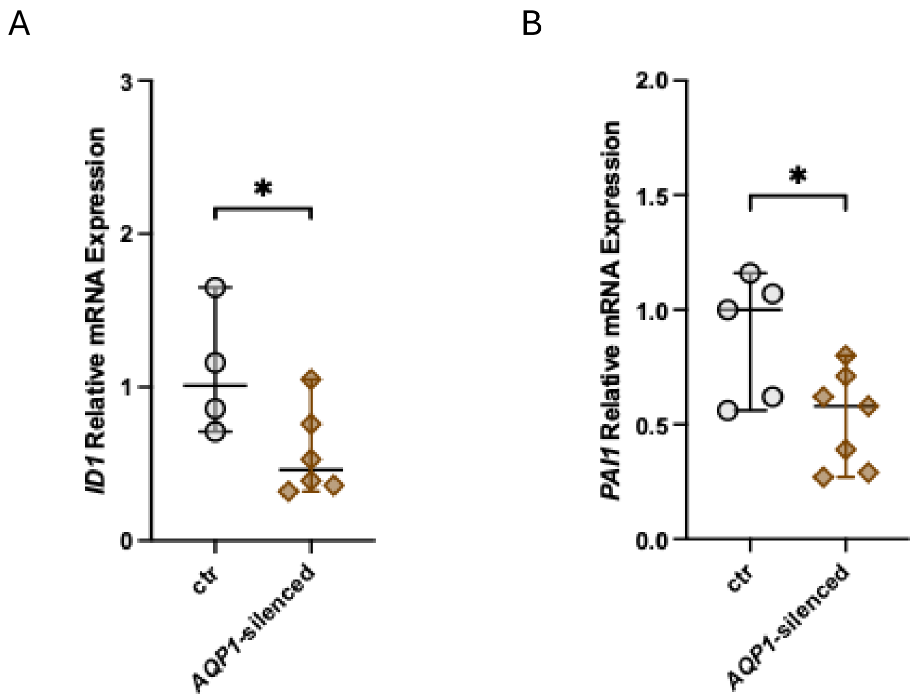

Figure S1. Effects of *AQP1* gene silencing on downstream molecules of the BMP/TGF-β signaling pathway in human pulmonary microvascular endothelial cells. HPMECs were silenced for the *AQP1* gene. The relative mRNA expression of the *ID1* (A), and *PAI1* (B) genes were estimated. Relative mRNA expression is shown as individual values (line in the middle, median values; whiskers, range of values) (A, n= 6; B, n= 7). The data presented are from at least four independent experiments. Transfection specificity (siRNA negative control) and efficiency (*AQP1* siRNA) were tested each time to ensure the consistency and reproducibility across the independent experiments. Statistical analysis was performed using the Student's t-test. *, $p < 0.05$ compared to non-transfected control HPMECs.
