# Supplementary material for: Disrupting BMP/TGF‐β Signaling: Modulation of AQP1 and TGFB1 in Human Pulmonary Microvascular Endothelial Cells
Source: Compr Physiol. 2025 Oct 29;15(6):e70066. doi: 10.1002/cph4.70066 (PMC12570780; doi:10.1002/cph4.70066)
Supplement: Supplementary file 2 — Figure S2: cph470066‐sup‐0002‐FigureS2.pdf. [file CPH4-15-e70066-s001.pdf]

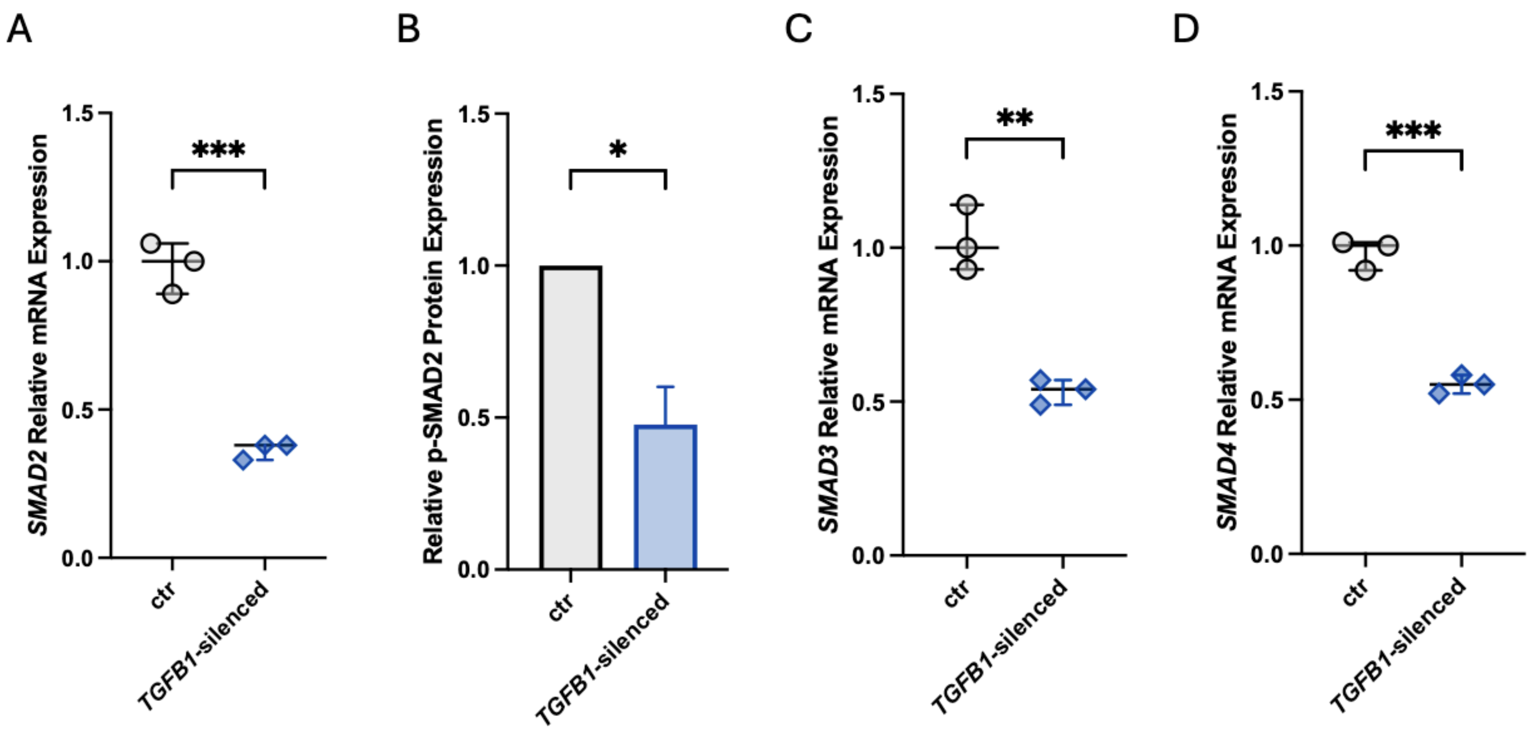

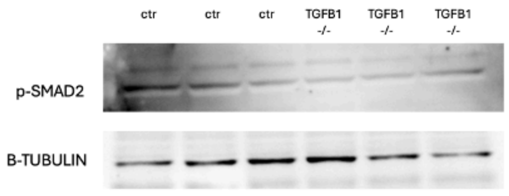

Figure S2. Effects of *TGFB1* gene silencing on downstream molecules of the BMP/TGF-β signaling pathway in human pulmonary microvascular endothelial cells. HPMECs were silenced for the *TGFB1* gene. The relative mRNA expression of the *SMAD2* (A), *SMAD3* (C), and *SMAD4* (D) genes were estimated, as well as the relative protein expression of pSMAD2 (D). Relative mRNA expression is shown as individual values (line in the middle, median values; whiskers, range of values) (A, n= 3; C, n= 3; D, n= 3). Protein expression was analysed by SDS-PAGE and immunoblotting, and relative expression was estimated by densitometry using β -tubulin as a loading control. Relative protein expression is shown with bar plots (mean ± SEM) (B, n= 3). The data presented are from three independent experiments. Transfection specificity (siRNA negative control) and efficiency (*TGFB1* siRNA) were tested each time to ensure the consistency and reproducibility across the independent experiments. Statistical analysis was performed using the Student's t-test. *, $p < 0.05$; **, $p < 0.01$; ***, $p < 0.001$ compared to non-transfected control HPMECs.
